# A New Method for Calculating the Relative Permeability Curve of Polymer Flooding Based on the Viscosity Variation Law of Polymer Transporting in Porous Media

**DOI:** 10.3390/molecules27123958

**Published:** 2022-06-20

**Authors:** Wenchao Jiang, Zhaowei Hou, Xiaolin Wu, Kaoping Song, Erlong Yang, Bin Huang, Chi Dong, Shouliang Lu, Liyan Sun, Jian Gai, Shichun Yao, Yunchao Wang, Chunlin Nie, Dengyu Yuan, Qinghua Xu

**Affiliations:** 1 Exploration and Development Research Institute, Petrochina Daqing Oilfield Company Limited, Daqing 163712, China; jiangwenchao@petrochina.com.cn (W.J.); houzhw@petrochina.com.cn (Z.H.); lushouliang@petrochina.com.cn (S.L.); gaijian@petrochina.com.cn (J.G.); yaoshch@petrochina.com.cn (S.Y.); wangyunchao@petrochina.com.cn (Y.W.); niechunlin@petrochina.com.cn (C.N.); yuandengyu@petrochina.com.cn (D.Y.); xuqinghua1@petrochina.com.cn (Q.X.); 2 Research and Development Center of Sustainable Development of Continental Sandstone Mature Oilfield, Daqing 163712, China; 3 Ministry of Education Key Laboratory of Enhanced Oil and Gas Recovery, Northeast Petroleum University, Daqing 163318, China; yangerlong@nepu.edu.cn (E.Y.); huangbin@nepu.edu.cn (B.H.); dongchi@nepu.edu.cn (C.D.); sunliyan@nepu.edu.cn (L.S.); 4 Daqing Oilfield Company Limited, Daqing 163453, China; wuxldq@petrochina.com.cn; 5 Unconventional Oil and Gas Science and Technology Research Institute, China University of Petroleum-Beijing, Beijing 102249, China

**Keywords:** hydrophobically associating polymer, relative permeability curve, polymer flooding, viscosity variation law, porous medium

## Abstract

Relative permeability of polymer flooding plays a very important role in oil field development. This paper aimed to measure and calculate the relative permeability curves of polymer flooding more accurately. First, viscosity variation law of polymer in porous media was studied. Rock particles of different diameters and cementing agent were used to make artificial cores and hydrophobically associating polymer solutions were prepared for experiments. Polymer solutions were injected into the cores filled with crude oil and irreducible water. In the process of polymer flooding, produced fluid was collected at different water saturations and locations of the core. Polymer solutions were separated and their viscosities were measured. With the experimental data, the viscosity variation rule of polymer transporting in porous media was explored. The result indicates that the viscosity retention rate of polymer solutions transporting in porous media has power function relationship with the water saturation and the dimensionless distance from the core inlet. Finally, the relative permeability curves of polymer flooding were measured by unsteady state method and the viscosity variation rule was applied to the calculation of the relative permeability curves.

## 1. Introduction

**Figure 1:**
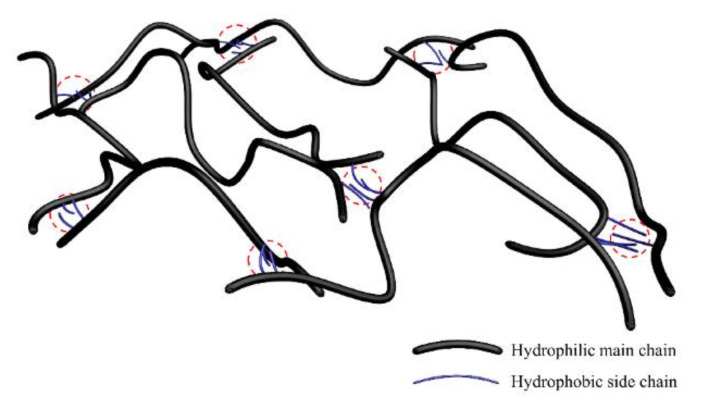
Three-dimensional schematic diagram of interactions of hydrophobically associating polymers.

As is known, polymer molecules in polymer solutions can increase the viscosity of water. For this reason, polymer is widely used in various kinds of oilfield production. There are different kinds of polymer used in oilfields, including partially hydrolyzed polyacrylamide (HPAM), xanthan gums, cellulosic, polyacrylate copolymers, etc. Additionally, HPAM is the most widely selected among them. However, as shielding of carboxyl groups weakens repulsive force and causes chain contraction, the salt resistance of HPAM is unsatisfactory. Furthermore, high temperature will affect the viscosity performance of HPAM. In addition, shearing action makes a big contribution to the HPAM chains’ mechanical degradation, which results in the decrease of the viscosity. Although xanthan gums are not easy to have shear degradation problems and can work well in relatively high salinity environment, high cost and poor injectivity restrict the xanthan gums’ wide application. Therefore, hydrophobically associating polymers (HAP), as a new kind of polymer, has been attracting more and more public attentions. HAP is synthesized by combining some hydrophobic side chains to a hydrophilic main chain [1] (Figure 1). Many studies have reported the synthesis and properties of HAP and results show that HAP has strong salt resistance. This is because salt addition has significant anti-electrolyte effect on zwitterionic polymer solutions and it can also enhance the association of zwitterionic polymers [2]. Furthermore, when the concentration of polymer solution is higher than critical association concentration, hydrophobic groups will have the tendency to form a transient reversible three-dimensional supramolecular network structure, which will make HAP have good thickening characteristics under high-temperature and high-salt conditions [3]. Due to the above characteristics, HAP is widely used in the field of oil development, such as hydraulic fracturing fluid preparation [4,5,6] and polymer flooding [7,8,9,10]. In this paper, HAP was selected to fit the high-temperature and high-salt condition of the Bohai Oilfield reservoir.

Relative permeability curves can describe the seepage state of multiphase fluid in porous media and play important roles in many aspects, such as history matching and the design of oil field development schemes. Studies on oil–water relative permeability [11,12,13,14,15,16,17,18,19,20,21,22] and oil–gas relative permeability [23,24,25,26,27] were carried out in recent years. For the acquisition of oil–water relative permeability curves, except that a few studies used some parameters to predict [16,17,18], most of them were measured by experiments [19,20,21,22]. Moreover, many researchers focused on the study of polymer flooding relative permeability curves. Experiments were conducted with the J.B.N. method [28] to get polymer flooding relative permeability curves, and results suggest that the water relative permeability decreases with the addition of polymer whether the cores are water-wet or oil-wet. Combining the Levenberg–Marquardt algorithm and the polymer-flooding numerical-simulation model, an inversion method of polymer flooding relative permeability curves was put forward [29,30]. The results, which are similar to the above studies, show that, compared with water flooding, water relative permeability of polymer flooding has a big drop. However, the precision of the inversed method depends heavily on that of polymer parameters. In addition, a study explored the influence of pore-scale polymer microspheres on the oil–water phase permeability and came to the conclusion that the addition of PMs makes the water phase relative permeability fluctuate obviously and the oil phase relative permeability increases first and then decreases [31].

When relative permeability curves of polymer flooding were measured and calculated by experiments, the viscosity variation rule of non-Newtonian fluid on the water relative permeability was of great importance and should be considered in the calculation. However, none of the above studies paid enough attention to this aspect. Some literature studied the mechanism and characteristics of polymer solution [32,33,34,35,36,37,38,39], especially HAP [40,41,42,43], seeping in porous media. However, no research described the viscosity of polymer solutions in different core positions and water saturations quantitatively.

Most physical simulations of polymer viscosity in porous media were carried out in cores without crude oil. Oil and polymer interact with each other during transportation in porous media. In addition, rock in the core has different actions on polymer molecules when there is oil in porous media or not. All these factors affect the viscosity of polymer solutions in porous media. As a result, the experiments above cannot simulate the rule of viscosity variation in the state of oil–water two-phase co-seepage in porous media to a greater extent. Moreover, current studies about the viscosity variation rule cannot describe the viscosity of polymer solutions concretely in different core positions and water saturations, which should be used in the calculation of the polymer flooding relative permeability curves to make the results more accurate.

In order to simulate the actual reservoir where oil and water exist simultaneously, the experiments of viscosity variation rule in this paper were conducted in the cores saturated with both water and crude oil. To deeply explore the viscosity variation trend, the viscosity of polymer solutions from different core positions was tested. Meanwhile, the viscosity of the polymer solutions at different core water saturations was examined. Finally, the results of the viscosity variation rule were used in the calculation of the water relative permeability curves of polymer flooding and more accurate relative permeability curves were obtained.

## 2. Experimental Materials and Methods

### 2.1. Experimental Materials

**Figure 2:**
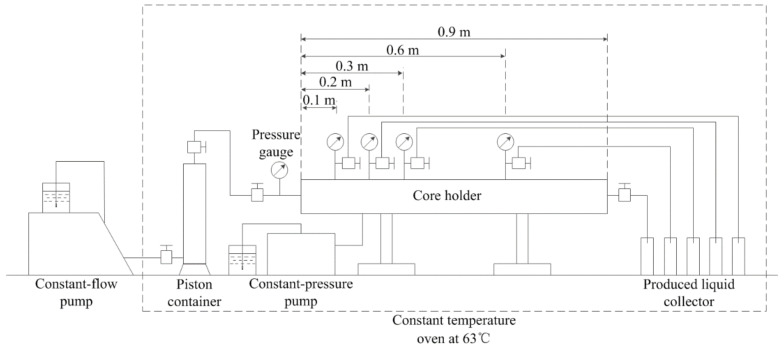
Experiment-process diagram.

The polymer employed in the experiments was a kind of hydrophobically associating polymer (HAP), which was provided by Sichuan Guangya Co., Ltd. (Nanchong, China). The polymer was composed of acrylamide, sodium acrylate and allyl cationic hydrophobic monomer. The molar ratio of three monomers was 80:20:0.2 and the hydrophobic monomer mole content was 0.20%. The molecular mass was about 1600 × 10^4^ and the degree of hydrolysis was 24.6%. The crude oil was got from Block X in Offshore Oilfield and its density was 950 kg/m^3^. The synthetic crude oil was made up of crude oil and aviation kerosene at a certain ratio to make the viscosity 70 mPa s at 63 °C. Artificial cores used in experiments were made of rock particles of different diameters, bentonite and cementing agent at a certain ratio. The permeability was about 2.5 μm^2^, and the porosity was about 30%. The size of the cores was 0.045 × 0.045 × 0.9 m, and all of the cores used in the experiments had extremely similar physical properties. A long core holder was used to hold cores. At 0.1, 0.2, 0.3 and 0.6 m away from the inlet of the core holder, there were outlets connected to a pressure gauge and a sample port. A constant-flow pump was used to push the polymer solution into the core at a stable speed. A constant-pressure pump was used to keep the pressure around the core constant. The experimental process was shown as follows (Figure 2).

The total dissolved solid in the water for experiments was 8552.56 mg/L. Composition of ion is exhibited in Table 1.

### 2.2. Experimental Methods

#### 2.2.1. Viscosity Measurement

The viscosity of the polymer solutions was measured on Brookfield DV- Ⅲ digital display viscometer. In order to simulate the effect of injection process on the polymer viscosity, the polymer solution was pre-cut by the following method:

The polymer solution was sheared by the No. 0 rotor for 30 s at the speed of 100 rpm. The instrument was preheated immovably for 90 s before the solution viscosity was tested. Measurements were done with the No. 0 rotor and the rotate speed was 6 rpm. The experimental temperature was 63 °C.

#### 2.2.2. Experiment Procedure of Viscosity Variation Experiment

1. The target concentration polymer solutions were prepared with the polymer HNT-300 and synthetic water. The apparent viscosity of the polymer solution was measured before being injected into the core.
2. The air in the core was extracted by a vacuum pump, and then the synthetic water was pushed into the pore in the core. The pore volume was equal to water volume minus the dead volume.
3. The synthetic crude oil was continually injected into the core at a constant injection rate and all outlets of the core holder were closed except the last one, which was farthest from the inlet. The injecting process lasted until that no more water was coming from the exit end. The steady pressure of the core holder inlet during the injecting process was recorded. The oil volume in the core was the same as the volume of the water produced from the core. The inlet and the last outlet were closed after this step to maintain the pressure field in the core.
4. The core with initial oil and irreducible water was placed at 63 °C for 12 h before use.
5. The polymer solution was injected into the core at a constant injection rate. In this process, the inlet of core holder was opened as soon as the pressure of inlet reached the steady pressure while injecting oil. From the water saturation reaching about 60%, samples were taken from each sampling port for every 0.05% increase in water saturation.

Before sampling, all outlets of the core holder were shut down after pressure values were obtained from the pressure gauges, which were connected with the sampling ports. Sampling was conducted from the sampling port close to the inlet to the ones far from the inlet. After one sample was collected, the pressures were restored to the values before sampling and then did the sampling from the next port.

6. The volumes of oil and water in the sample were measured as soon as the sample was obtained. Then, the water was separated from the sample. Next, the viscosity of the produced water was measured at once.

#### 2.2.3. Experiment Procedure of Polymer Flooding Relative Permeability

Relative permeability curves were measured by unsteady state method and the preliminary steps of the experiment were the same as the No. 1 to No. 4 steps of viscosity variation experiment procedure. However, the difference between the two experiments was that, in the polymer flooding relative permeability experiment, no sampling was done from the middle four ports of core holder and the sample was only got from the last exit end. Meanwhile, the viscosity did not need to be paid attention to and only the pressure of the inlet and the volumes of the produced oil and water were measured and recorded.

#### 2.2.4. Calculation Method of Relative Permeability Curve

J.B.N. method was used to process experimental data and calculate relative permeability curves. The formulae used were as follows [28]:

(1) $$f_{o}\left(S_{w}\right)=\frac{d{\bar{V}}_{o}\left(t\right)}{d\bar{V}\left(t\right)}$$

(2) $$K_{\mathrm{ro}}=f_{o}\left(S_{w}\right)\times \frac{d\left[1/\bar{V}\left(t\right)\right]}{d\left[1/I\bar{V}\left(t\right)\right]}$$

(3) $$K_{\mathrm{rw}}=K_{\mathrm{ro}}\times \frac{\mu_{w}}{\mu_{o}}\times \frac{1-f_{o}\left(S_{w}\right)}{f_{o}\left(S_{w}\right)}$$

(4) $$I=\frac{Q\left(t\right)}{Q_{o}}\times \frac{\Delta p_{o}}{\Delta p\left(t\right)}$$

(5) $$S_{\mathrm{we}}=S_{\mathrm{wi}}+{\bar{V}}_{o}\left(t\right)-\bar{V}\left(t\right)f_{o}\left(S_{w}\right)$$

where *f*_o_(*S*_w_) is oil content, *S*_w_ is water saturation of the core, ${\bar{V}}_{o}\left(t\right)$ is dimensionless cumulative oil production, $\bar{V}\left(t\right)$ is dimensionless cumulative liquid production, *K*_ro_ is relative permeability of oil phase, *I* is relative injection capacity, *K*_rw_ is relative permeability of water phase, *μ*_w_ is viscosity of synthetic water, *μ*_o_ is viscosity of synthetic crude oil, *Q*(*t*) is liquid production at the outlet of core at time t when constant speed injection is used, *Q*_o_ is oil production at the outlet of core at the initial time, Δ*p*_o_ is driving differential pressure at the initial time, Δ*p*(*t*) is driving differential pressure at time *t*, *S*_we_ is water saturation of the outlet, *S*_wi_ is irreducible water saturation.

## 3. Results and Discussion

### 3.1. Results and Discussion of the Viscosity Variation

#### 3.1.1. Experimental Results and Data Analysis

**Figure 3:**
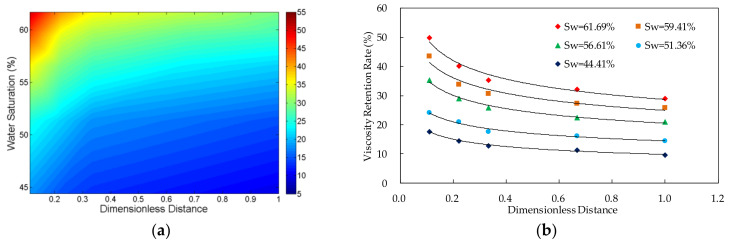
The relationship between viscosity retention rate of polymer solution, dimensionless distance and water saturation in the experiment VV-1 in which the polymer concentration is 500 mg/L. (**a**) Contour map of viscosity retention rate; and (**b**) scatter plot of viscosity retention rate.

**Figure 4:**
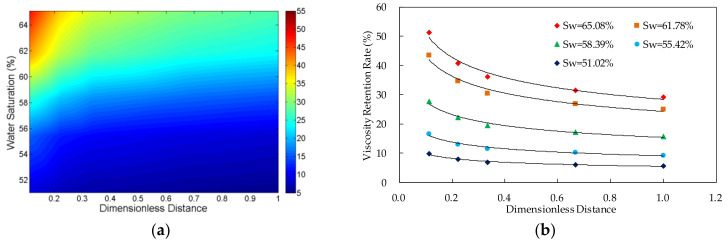
The relationship between viscosity retention rate of polymer solution, dimensionless distance and water saturation in the experiment VV-2 in which the polymer concentration is 1250 mg/L. (**a**) Contour map of viscosity retention rate; and (**b**) scatter plot of viscosity retention rate.

**Figure 5:**
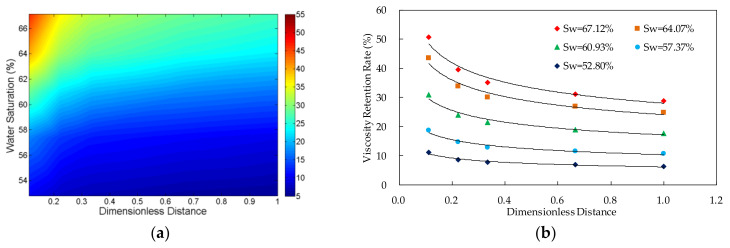
The relationship between viscosity retention rate of polymer solution, dimensionless distance and water saturation in the experiment VV-3 in which the polymer concentration is 1750 mg/L. (**a**) Contour map of viscosity retention rate; and (**b**) scatter plot of viscosity retention rate.

**Figure 6:**
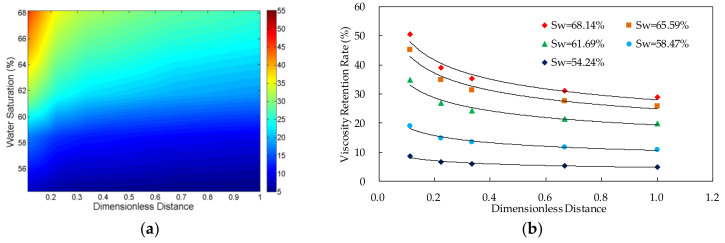
The relationship between viscosity retention rate of polymer solution, dimensionless distance and water saturation in the experiment VV-4 in which the polymer concentration is 2000 mg/L. (**a**) Contour map of viscosity retention rate; and (**b**) scatter plot of viscosity retention rate.

The viscosity retention rate (VRR) of the polymer solutions in different core locations and different water saturations was shown in Figure 3, Figure 4, Figure 5 and Figure 6. The detailed experimental schemes were displayed in Table 2.

From the scatter plot of the dimensionless distance and the viscosity retention rate, it can be found that there is an approximate power function relationship between them. The parameters were fitted by power function. The results in Table 2 indicate that the significance values of all fitting functions are below 0.01 and the null hypothesis is rejected, which suggests that the functions are statistically significant. Furthermore, the values of determination coefficients of all the functions are above 0.9, which indicates that the fitting functions are highly accurate. The two parameters can be expressed by Formula (6):

*VRR*(*S*_w_,*L*_d_) = *a* × *L*_d_*^b^* (6)

where *L*_d_ is dimensionless distance from core inlet, *VRR*(*S*_w_,*L*_d_) denotes viscosity retention rate of polymer solutions when the water saturation is *S*_w_ and the dimensionless distance is *L*_d_, *a* and *b* denote the fitting coefficient relating to concentration of polymer solution and water saturation.

The information of fitting models is shown in Table 2. As the values of Coefficient *b* were negative and the value of *VRR* could not be greater than 100%, the value of *L*_d_ should be greater than the minimum value which make the *VRR* value 100%.

**Figure 7:**
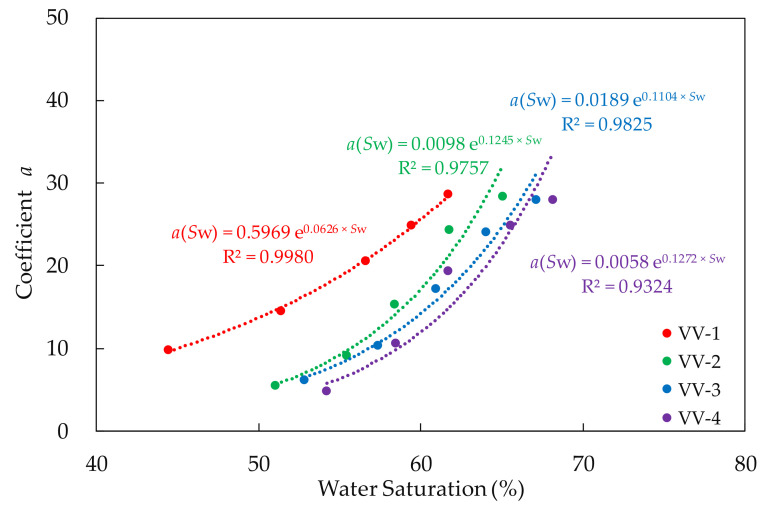
The relationship between the coefficient *a* in Formula (7) and water saturation.

As is shown in Table 2, the values of the coefficient *b* in the formulae of different experiments are all in the range of −0.275 to −0.231 and concentrated around −0.24. Therefore, the average value of −0.248 is determined as the value of coefficient *b*. The coefficient *a* is related to water saturation and polymer concentration in the system. Furthermore, from the scatter plot of the water saturation and the coefficient *a* in Figure 7, it can be found that the two coefficients have the power function relationship, which can be expressed as Formula (7):

*a*(*S*_w_) = *m* × e*^n^*
^× *S*w^ (7)

where *m*, *n* denote fitting coefficient relating to concentration of polymer solution.

The coefficient *m* and *n* in the formula are related to the concentration of polymer solution. Additionally, the information of fitting models is shown in Table 3.

As is shown in Table 3, the significance values of all functions are all below 0.01 and the null hypothesis is rejected, which suggests that the functions are statistically significant. In addition, the values of the determination coefficients of the models are all above 0.9, which indicates that the fitting functions are of high accuracy. Furthermore, if Formula (7) is brought into Formula (6), a new relationship can be obtained to describe the viscosity retention rate of polymer solution transporting in the core. The function can be expressed as Formula (8):

*VRR*(*S*_w_,*L*_d_) = *m* × e*^n^*
^× *S*w^ × *L*_d_^−0.248^ (8)

As the viscosity retention rate can be defined as:

(9) $$VRR\left(S_{w},\;L_{d}\right)=\frac{\mu \left(S_{w,}L_{d}\right)}{\mu_{a}}$$

where *μ*(*S*_w_,*L*_d_) denotes the viscosity of polymer solutions when the water saturation is *S*_w_ and the dimensionless distance is *L*_d_, *μ*_a_ is apparent viscosity of polymer solution.

The viscosity of polymer solution transporting in cores was described as Formula (10):

*μ*(*S*_w_,*L*_d_) = *μ*_a_ × *m* × e*^n^*
^× *S*w^ × *L*_d_^−0.248^ (10)

#### 3.1.2. Relationship between Viscosity Retention Rate and Dimensionless Distance

The result shows that, in the case of the same water saturation, the viscosity of the polymer solution transporting in the porous media decreases with the increase of the distance from the inlet. Additionally, the viscosity loss of the system happens mainly near the inlet, especially the first third length of the core.

This is because the polymer molecules are blocked seriously near the inlet and the cross-section area allowing the polymer molecules to transport reduces. Thus, the hydrophobically associating polymers, which have three-dimensional network structure, are subjected to stronger shear and pull forces. This results in the fracture of the polymer network structures. As three-dimensional network structure of polymer makes great contributions to the viscosity increase of the polymer solution, the fracture of the network structure directly leads to the decrease of the solution viscosity. Additionally, some polymer molecules are absorbed by the porous media instead of transporting further in the porous media. Therefore, the viscosity of the solution in porous media highly decreases.

With the increase of the dimensionless distance from the inlet, the shear action becomes weaker, but still works. Furthermore, the absorption of polymer molecules by porous media also exists and contributes to the decrease of the solution viscosity.

#### 3.1.3. Relationship between Viscosity Retention Rate and Water Saturation

The experimental data indicates that, in the case of the same dimensionless distance, the viscosity retention rate of the polymer solution increases with the increase of the water saturation in the core. Furthermore, from the Formula (8), it can be found that when the value of the dimensionless distance is the same, the viscosity retention rate has power function relationship with the water saturation.

The result above suggests that the viscosity of the polymer solution produced from the core outlet increases with the increase of the water saturation. There are two main reasons for this phenomenon. Firstly, the polymer molecules are absorbed by the porous media and it results in the decrease of the solution viscosity. With the continuous injection of the solution, the polymer adsorption in the porous media gradually reaches saturation and the resulting decline rate of the viscosity decreases. Secondly, with the increase of the water saturation, there is less crude oil in the pore. Due to the reduction of residual oil such as membranous residual oil, the cross-section area, which allows the polymer molecules to transport, becomes larger. Thus, the shear and the pull force acting on the hydrophobically associating polymers with three-dimensional stereoreticular structure decreases. For these two reasons above, the viscosity retention rate of the polymer solution produced from the core outlets increases with the increase of the water saturation.

In addition, if the four experiments, using polymer solution of different concentration, are compared, the conclusion can be figured out that, in the case of the same dimensionless distance and similar water saturation, the viscosity retention rate decreases with the increase of the polymer solution concentration. This is mainly because the higher the concentration of polymer solution is, the stronger the network structure which is formed by cross-linking, the stronger the shear action in the core and the greater the viscosity loss.

### 3.2. Results and Discussion of the Polymer-Flooding Relative Permeability

#### 3.2.1. Calculation of the Relative Permeability Curves of Polymer Flooding

The procedure of viscosity variation experiment was very similar to relative permeability experiment. Moreover, the oil, water, polymer solution and cores used in two experiments were all the same. Hence, the viscosity change process in the relative permeability experiment was considered identical with the viscosity variation experiment. Based on the above, the formula of viscosity variation rule was added to J.B.N. method to describe the dynamic change of polymer solution viscosity in the polymer relative permeability experiment.

First, in order to express the average viscosity of polymer solution in whole core under a certain water saturation, definite integral of Formula (10) with respect to the *L*_d_ from *L*_min_, which was equal to the minimum value of *L*_d_, to 1 was calculated.

(11) $$\mu \left(S_{w}\right)=\int_{L_{\min}}^{1}\mu_{a}\times m\times e^{n\times S_{w}}\times L_{d}{}^{-0.248}dL_{d}$$

Average viscosity calculation results of polymer solution in whole core under different water saturation in each experiment were shown in Table 4. Apparent viscosity and viscosity of polymer solution from outlet were also exhibited for comparison.

**Figure 8:**
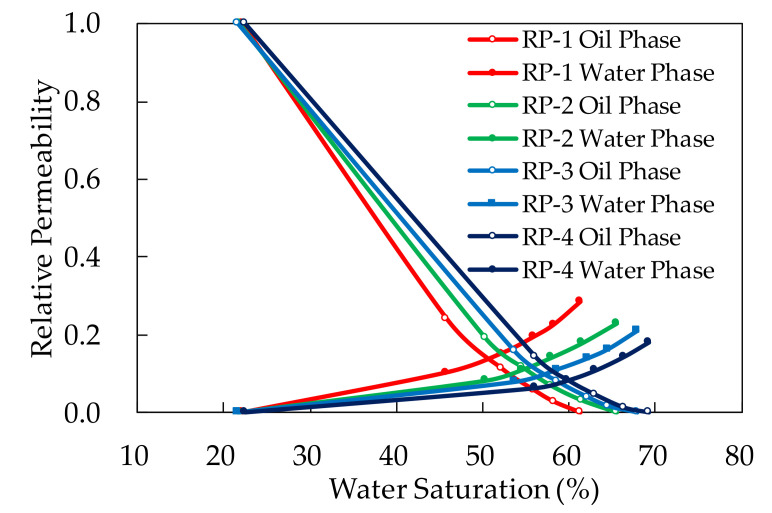
The relative permeability curves of polymer flooding calculated by the improved J.B.N. method.

Formula (11) was added to Formula (3) and *K*_rw_ was expressed as Formula (12):

(12) $$K_{\mathrm{rw}}=K_{\mathrm{ro}}\times \frac{\int_{L_{\min}}^{1}\mu_{a}\times m\times e^{n\times S_{w}}\times L_{d}{}^{-0.248}dL_{d}}{\mu_{o}}\times \frac{1-f_{o}\left(S_{w}\right)}{f_{o}\left(S_{w}\right)}$$

with the improved J.B.N. method above, the polymer flooding relative permeability curves were calculated and shown in Figure 8.

**Figure 9:**
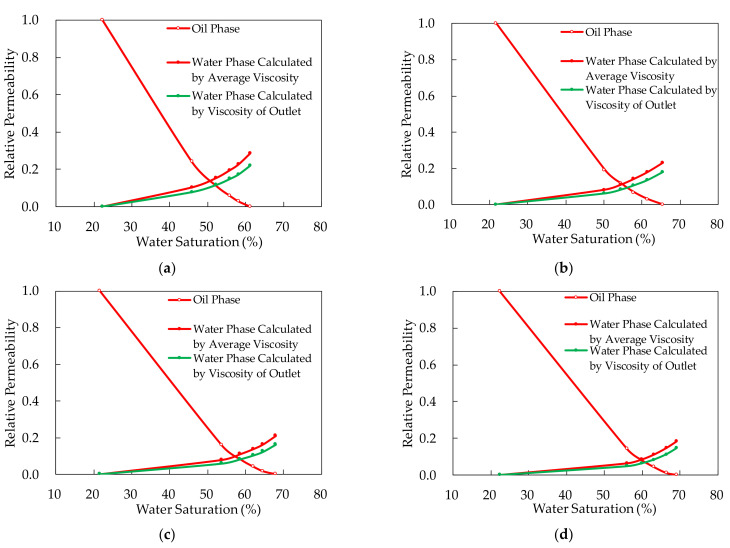
Polymer solution relative permeability curves calculated by different viscosity methods. (**a**) Experiment RP-1 in which the polymer concentration is 500 mg/L; (**b**) Experiment RP-2 in which the polymer concentration is 1250 mg/L; (**c**) Experiment RP-3 in which the polymer concentration is 1750 mg/L; (**d**) Experiment RP-4 in which the polymer concentration is 2000 mg/L.

Polymer solution relative permeability curves calculated by different viscosity methods were shown in Figure 9.

#### 3.2.2. Analysis of Polymer Relative Permeability Curves Calculated by Different Viscosity Methods

1. The integral viscosity can express the average viscosity of the whole core to the maximum extent. Thus, the polymer relative permeability curves calculated by the integral average viscosity are more accurate than others. From the experimental results, it can be figured out that the polymer solution relative permeability calculated by viscosity of polymer solution from the outlet was lower than that calculated by integral viscosity.
2. Comparing the relative permeability curves of polymer flooding with different concentrations, we can find that, with the increase of polymer concentration, the relative permeability of water phase decreases and that of oil phase increases. The reduction of water phase permeability is due to the polymer retention in pore, which is caused by adsorption, mechanical capture and hydrodynamic capture. Furthermore, polymer molecules also have strong hydrogen bond with water molecules, which makes the adsorption of water molecules on the adsorption layer stronger. These two reasons explain the decrease of water phase relative permeability. Additionally, for the increase of oil phase, polymer molecules do not hinder the flow of oil. Furthermore, the adsorption layer formed by polymer molecules on the pore wall of rock will make the wall surface of pore throat smoother, which will reduce the flow resistance of oil.
3. From the curves, it can also be figured out that the higher polymer concentration, the lower residual oil saturation. Polymer molecules can improve the mobility of water and increase the micro sweep efficiency. As a result, the increase of polymer concentration can reduce the residual oil saturation in the core.

## 4. Conclusions

1. The viscosity retention rate of the polymer solution transporting in the core is related to the dimensionless distance from the inlet and the water saturation. The relationship can be expressed by the power function formula: *VRR*(*S*_w_,*L*_d_) = *m* × e*^n^* × *S*_w_ × *L*_d_^−0.248^
2. In the case of the same water saturation, viscosity of the polymer solution transporting in the porous media decreases with the increase of the distance from the inlet and the viscosity loss happens mainly near the inlet, especially the first third length of the core. Additionally, the two coefficients have power function relationship. Meanwhile, in the case of the same dimensionless distance, the viscosity of the polymer solution transporting in porous media increases with the increase of the water saturation in the core and the two coefficients have power function relationship.
3. The viscosity calculated by the integral formula is more representative of the average viscosity of polymer solution in the core, and the relative permeability curves of polymer flooding calculated by this viscosity are more able to describe the actual situation.
4. With the increase of polymer concentration, the relative permeability of oil phase increases, and the relative permeability of water phase and the residual oil saturation decrease.

## Figures and Tables

**Table 1:** Composition of ion in the synthetic water.

**Ionic** **Composition**	Na^+^	K^+^	Ca^2+^	Mg^2+^	Cl^−^	CO_3_^2−^	HCO_3_^−^	SO_4_^2−^	Total Salinity
**Concentration** **(mg/L)**	2819.78		270.01	111.04	4791.47	54.01	468.17	38.08	8552.56

**Table 2:** Summary, ANOVA and coefficients of Formula (6) (Models are calculated by SPSS).

Experiment Number	Polymer Concentration (mg/L)	Water Saturation (%)	R^2^	Sig.	Coefficient *a*	Coefficient *b*	Minimum Value of *L*_d_
VV-1	500	44.41	0.99	0.000	9.819	−0.265	7.95 × 10^−5^
		51.36	0.98	0.001	14.451	−0.232	4.59 × 10^−4^
		56.61	0.99	0.001	20.531	−0.238	1.73 × 10^−3^
		59.41	0.96	0.004	24.924	−0.231	3.50 × 10^−3^
		61.69	0.98	0.002	28.671	−0.238	6.23 × 10^−3^
VV-2	1250	51.02	0.99	0.001	5.438	−0.253	9.10 × 10^−6^
		55.42	0.98	0.001	9.067	−0.259	8.29 × 10^−5^
		58.39	0.98	0.001	15.377	−0.255	3.68 × 10^−4^
		61.78	0.98	0.001	24.291	−0.25	2.02 × 10^−3^
		65.08	0.98	0.001	28.403	−0.254	1.06 × 10^−2^
VV-3	1750	52.8	0.97	0.002	6.161	−0.25	1.56 × 10^−5^
		57.37	0.97	0.003	10.376	−0.249	1.19 × 10^−4^
		60.93	0.97	0.003	17.16	−0.247	5.83 × 10^−4^
		64.07	0.97	0.003	24.094	−0.249	2.36 × 10^−3^
		67.12	0.97	0.002	28.023	−0.249	9.16 × 10^−3^
VV-4	2000	54.24	0.95	0.004	4.792	−0.243	9.98 × 10^−6^
		58.47	0.97	0.002	10.604	−0.245	8.74 × 10^−5^
		61.69	0.97	0.003	19.336	−0.246	4.56 × 10^−4^
		65.59	0.96	0.003	24.866	−0.25	3.37 × 10^−3^
		68.14	0.97	0.002	27.979	−0.247	1.25 × 10^−2^

**Table 3:** Summary, ANOVA and coefficients of Formula (7) (Models are calculated by SPSS).

Experiment Number	Polymer Concentration(mg/L)	R^2^	Sig.	Coefficient *m*	Coefficient *n*
VV-1	500	0.998	0.000	0.5969	0.0626
VV-2	1250	0.976	0.002	0.0098	0.1245
VV-3	1750	0.983	0.001	0.0189	0.1104
VV-4	2000	0.932	0.008	0.0058	0.1272

**Table 4:** Average viscosity calculated by integral formula and other viscosities.

Experiment Number	Polymer Concentration (mg/L)	Water Saturation (%)	Average Viscosity (mPa·s)	Viscosity of Polymer Solution from Outlet (mPa·s)	Apparent Viscosity (mPa·s)
RP-1	500	45.81	0.9	0.7	6.2
		52.13	1.3	1.0	
		55.89	1.6	1.2	
		58.19	1.9	1.4	
		61.28	2.2	1.7	
RP-2	1250	50.28	1.5	1.1	21.6
		54.61	2.5	1.9	
		57.96	3.8	2.9	
		61.46	5.9	4.5	
		65.53	9.4	7.4	
RP-3	1750	53.68	3.7	2.8	39.5
		58.59	6.4	4.8	
		62.08	9.3	7.1	
		64.59	12.2	9.3	
		67.89	17.2	13.4	
RP-4	2000	56.03	4.7	3.5	48.6
		59.81	7.5	5.7	
		63.02	11.3	8.5	
		66.36	17.0	13.1	
		69.18	23.5	18.7	

## Data Availability

Not applicable.

## Sample Availability

Samples of the compounds are not available from the authors.
